# Thermoacoustic Sandwich Panels Produced with Balsawood or Pineapple Fiber as Core and *Gmelina arborea* Wood as External Veneer

**DOI:** 10.1021/acsomega.5c02267

**Published:** 2025-07-02

**Authors:** Andres Villalta-Céspedes, Aldo Joao Cárdenas-Oscanoa, Markus Euring, Roger Moya

**Affiliations:** 1 Escuela de Ingeniería Forestal, 27942 Instituto Tecnológico de Costa Rica, Cartago Apartado 159-7050, Costa Rica; 2 Department of Wood Technology and Wood-Based Composites, Georg-August University Goettingen, Goettingen 37073, Germany; 3 Forest Industry Department, Faculty of Forest Sciences, 113018 Universidad Nacional Agraria La Molina, Lima 15024, Perú

## Abstract

The utilization of composite sandwich panels (CSP) with a core composed of wood or natural fibers presents a sustainable option for building insulation to address climate change. This study aims to produce and assess CSP thermoacoustic insulators by examining their physical, mechanical, acoustic, and thermal characteristics. The panels, with thicknesses of 12 and 19 mm, are constructed using cores of balsawood or pineapple leaves (*Ananas comosus*) (PALF) variety M2 and melina wood (*Gmelina arborea*) as veneer. Findings indicate that the density of the panels was from 222 to 266 kg m^–3^ for CSP-balsawood and from 210 to 303 kg m^–3^ for CSP-PALF. Regarded water absorption panel values, for CSP-balsawood is between 60 and 69% while for CSP-PALF, it is between 104 and 128%. Swelling values of 0.92–1.53 and 3.4–8.5% are for CSP-balsawood and CSP-PALF, respectively. The CSP-balsawood demonstrated superior modulus of rupture and modulus of elasticity values in static bending in both longitudinal and parallel directions, as well as in compression and tension. Furthermore, with the same core material, the 19 mm CSP exhibited greater density and mechanical properties compared to the 12 mm CSP. Thermal resistance is 0.26 to 0.30 and 0.19 to 0.25 W m^–1^ K^–1^ for panels from balsawood and PALF, respectively, with the highest thermal conductivity observed at a 19 mm thickness in both cases. During sound absorption testing, the SAC coefficient was found to be less than 0.33 sound absorption coefficient for different panels, which is characteristic of insulation panels. Results reported that CSP-balsawood is suitable for applications where sound insulation is a priority, whereas those made with PALF are more appropriate when thermal insulation is the primary concern. The fabrication of CSP with natural products enhances energy efficiency, improves spatial conditions, and decreases energy consumption, thereby contributing to climate change mitigation.

## Graphical abstract

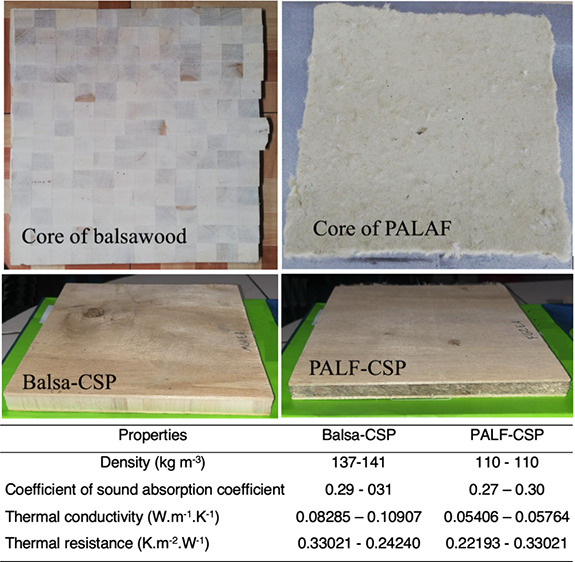

## Introduction

Nowadays, numerous sectors have been recognized as contributors to greenhouse gas emissions, prompting each to explore various strategies to mitigate these impacts.^1^ Specifically, the construction industry stands out as a significant consumer of energy worldwide.^2^ This industry is estimated to be responsible for approximately 36% of all emissions.^3^ Consequently, there is a strong emphasis within this field on utilizing materials derived from renewable engineering sources to protect the environment and diminish the reliance on synthetic, petroleum-based materials and other nonrenewable resources that demand high energy levels for construction.^2^ Additionally, fluctuations in building temperatures adversely affect the quality of life, thermal comfort, and productivity of individuals, while also increasing the demand for energy, such as electricity and water.^4^ Furthermore, there is a focus on materials with low sound transmission due to the rise in the construction of multifunctional buildings and multifamily residences.^5^

Composite sandwich panels (CSP) have recently experienced increased usage due to their ability to be produced from various renewable resources.^6^ These panels are lightweight and serve as multifunctional engineering structures, created by placing a core material between two thin, rigid outer shells.^6,7^ CSP consist of two exterior layers made from metals, plastics, or other stiff materials, which are separated by a central component typically composed of foams, panels, cellular structures, or lightweight substances such as wood or nonwood fibers.^8,9^

Wood-based and nonwood CSP are increasingly utilized in construction because of their environmental benefits and effectiveness within the industry and various other sectors.^10^ These CSP are constructed in layers, which enhances their mechanical strength, offers excellent thermal insulation, and reduces weight.^6^ This construction material relies on renewable resources^11^ and addresses issues related to the excessive use of harmful adhesives by incorporating adhesives derived from natural sources.^10^ This shift encourages the use of natural resources and cuts down on greenhouse gas emissions.^11^ Nonwood-based CSP, which have been developed more recently, primarily utilize fibers sourced from waste generated by other processes. They have been introduced to expand the options available for manufacturing eco-friendly panels and to enhance the ecological impact of other processes,^9^ offering an alternative to materials derived from petroleum.^8^

CSP were initially designed primarily for structural purposes. However, in recent times, these engineering products have found applications as thermal and acoustic insulation in buildings.^12^ This is due to their unique manufacturing process, which results in a lightweight composite made by placing a core between two thin, rigid layers.^6^ This design allows for the use of highly effective acoustic or thermal materials in the central part of the panel, while the outer layers provide structural support.^7^ Utilizing insulating panels made from renewable materials can eliminate the need for substances that are hazardous to human health and require significant energy for extraction and production, like cement and petroleum-based products.^8,9^ This approach also helps decrease the energy consumption needed to maintain buildings at a suitable temperature without losing heat.^12^

CSP made with a wood core feature a wide range of wood species.^11^ However, when it comes to producing low-density panels, the choice of species is more restricted. Balsawood (*Ochroma pyramidale*) stands out as a desirable option among sustainable materials for constructing CSP due to its impressive mechanical properties relative to its density.^13^ This makes it an appealing choice for the cores of such panels, with numerous applications in engineering products used across civil infrastructure (like windmills and bridges), transportation (including cars, trucks, caravans, trains, aircraft, and boats), industrial sectors (such as packaging and storage), and leisure activities (like sports equipment and musical instruments).^13–15^ Balsawood is a tropical species that grows naturally from the southern regions of Mexico to Bolivia, within latitudes of 22° N to 15° S.^13^ Recently, it has been cultivated under the concept of fast-growth plantations in tropical parts of the Americas and other tropical regions worldwide.^16^ The goal is to produce sawlogs as quickly as possible with suitable density.^17^ Its density is relatively low, ranging between 50 and 350 kg m^–3^, which varies with tree age.^14^

Conversely, Costa Rica holds the position of the global leading exporter of fresh pineapple, making this industry a significant source of jobs and revenue for the nation.^18^ Nevertheless, this sector faces substantial challenges, particularly concerning the environmental repercussions of its production processes and the handling of organic waste. Pineapple cultivation is notable for producing a considerable amount of waste after harvesting, as more than half of the weight of the plant is discarded.^19,20^ Consequently, there is an ample supply of pineapple stubble available as a raw material that can be utilized in innovative technologies, thereby supporting the circular economy by repurposing waste from the pineapple industry.^21^ Among the postharvest uses of pineapple stubble, the most significant is the extraction of pineapple leaf fiber (PALF), with a substantial quantity of this fiber being retrievable either from individual plants^22^ or from the dense plantation areas, which can range from 20,000 to 40,000 plants per hectare.^20^

These two materials are known for their diverse applications across various composite types. Balsawood, for instance, is predominantly utilized in composite sandwich panels (CSP) in both core- and outer-layer designs. Galos et al.^13^ provide an in-depth review of balsa’s role as a core material in sandwich panels, detailing its mechanical properties when combined with various other materials. However, their review offers limited insight into its thermal and acoustic characteristics. On the other hand, PALF is recognized as a fiber with extensive applications in composite reinforcement, showcasing remarkable versatility in its uses and the types of adhesives that can be employed to create composites.^23,24^

In this regard, creating PALF composites involves using a particular type of foam where the binder plays a crucial role, reaching up 8 to 12% of the total weight.^25^ Several binders are available, originating from synthetic sources, and more recently, some adhesives have been developed from more sustainable sources.^26^ Lately, polylactic acid (PLA) has gained attention as an eco-friendly thermoplastic polyester, as it is derived from the fermentation of agricultural products such as corn, starch, potatoes, sugar cane, beets, and other similar sources.^25^ As a result, PLA can be utilized in the production of foams for diverse applications, including those with acoustic and thermal properties.^27–29^

*Gmelina arborea* (melina) is widely used in commercial reforestation programs in tropical countries for sawn wood production, pulp, or bioenergy.^30^ *G. arborea* is one of the most important species of timber for solid wood production in Costa Rica, and its wood has been utilized in many composite products, such as plywood, cross-laminated timber, or fiber wood.^31^

Given the potential of Costa Rica for balsa wood production and the considerable waste from pineapple production, which holds promise for creating composites with more environmentally sustainable binders, the aim of this study is to produce and assess CSP in terms of its physical, mechanical, acoustic, and thermal characteristics. The focus is on sandwich core panels with thicknesses of 12 and 19 mm, made from low-density materials such as *Ochroma pyramidale* (balsawood) or *Ananas comosus* fiber (pineapple) variety M2 for the core, and *Gmelina arborea* (melina wood) veneer for the outer layers. We hope that the insulation panels fabricated with these materials present appropriate thermal and acoustic properties.

These findings demonstrate the technical feasibility of such sandwich panels, enabling their application in various systems to enhance energy efficiency, improve spatial conditions, and decrease energy consumption, thereby contributing to climate change mitigation.

## Materials and Methods

### Materials

In producing CSP, two distinct core materials were utilized in the core part: *Ochroma pyramidale* wood pieces, commonly known as balsawood, bonded with polyvinyl acetate (PVA), and PALF bonded with polylactic acid (PLA). The outer surfaces of the panels were finished with veneers of *Gmelina arborea* (melina wood), which were adhered to the core using PVA adhesive. The balsawood was sourced from a rapidly growing plantation that was 4.5 years old, located in Guácimo de Limón, Costa Rica (10°52′41″ N, 84°21′88″ W). The sawlogs had diameters ranging between 14 and 18 cm. PALF was derived from the leaves of *Ananas comosus* (pineapple) plants of variety M2, collected from monocultures in the community of Pinar in the Pital district of San Carlos, Alajuela, Costa Rica (10°26′09 N and 84°14′58 W). The moisture content for balsawood was between 150 and 220%, while for PALF, it was between 90 and 95%.

Polylactic acid (PLA) is a bicomponent fiber, composed of two concentric layers, with a round cross section and sinuous shape, a fiber diameter between 14–16 μm, fiber fineness of 2.42 dtex, and fiber length of 6 mm. The PLA core melting point is 175 °C, while the sheath melting point is 130 °C. PLA was provided by Indorama Ventures Fibers Germany GmbH (Hattersheim, Frankfurt, Germany). PVA adhesive was provided by LANCO (San Jose, Costa Rica). The technical description of the product indicates that the resin is poly­(vinyl acetate) and water, presenting 54.5–55.5% solid content and 1600–2200 cP viscosity. No further modifications were made to these materials.

### Balsawood, Pineapple Leaves, and Melina Veneer Preproduction Process

The logs were sawn using a pattern commonly used by the Costa Rican furniture industry,^31^ in which the pieces obtained were 60 mm thick. This thickness was selected because the international market requires dry wood with a final thickness of 50 mm.^32^ The logs presented growth stress, so an initial cut was made to relieve these stresses; then, the log was turned over to rest it on its straight side and obtain 60 mm-thick boards in the variable width of the log. Then all of them were edged, ensuring that each one had four finished edges. Balsawood was dried using solar energy for 480 h and a moisture content target of 12%.^33^

In the case of the extraction of fibers from the leaves, the machine model proposed by Moya and Camacho^20^ was used. This machine was put to work by introducing 4–6 pineapple leaves tip first. The worker must hold the leaves from the base. Once half the length of the leaf has been introduced, it must be taken out backward. At this stage, the leaf fiber is separated from the parenchymal tissue. Later, the worker holds the leaves by the already shredded extreme and introduces them to their bases first until reaching the exposed fiber. Then the leaves are taken out, with the fiber completely extracted.

The veneer applied to the surfaces measured 3 mm and was provided by the company Maderas Cultivadas de Costa Rica S.A. This company procures the veneers from trees that are 10–12 years old, sourced from commercial plantations located across the northern region of Costa Rica. The sheets underwent a drying process in a solar oven until their moisture content was reduced to between 6 and 8%. Subsequently, 40 dried sheets with thicknesses of 12 and 19 mm were cut to a size of 62 cm by 62 cm. It was ensured that these sheets were free from knots, cracks, and any damage caused by stains or rot.

### Composite Sandwich Panel Production

Four types of CSP were produced, featuring two distinct sandwich core materials: balsawood (illustrated in Figure 1d) and PALF (depicted in Figure 1j). These panels were made in two different thicknesses: 12.7 and 19.1 mm (shown in Figure 2a) and were faced with two layers of 3 mm *G. arborea* veneers in all the cases. The treatments are categorized as Balsa-CSP-12, Balsa-CSP-19, PALF-CSP-12, and PALF-CSP-19 (Table 1). The study focused on the impact of varying the core type and thickness. In total, 20 panels were produced, with each treatment comprising five panels.

**Table 1:** Characteristics of Different CSP of Balsawood and PALF with Acoustic and Thermal Insulation Properties^a^

				adhesive utilized in	
core type	thickness of board sandwich (mm)	code	core thickness (mm)	core	veneer face
balsawood	12.7	Balsa-CSP-12	6.7	PVA	PVA
	19.1	Balsa-CSP-19	13.1	PVA	PVA
PALF	12.7	PALF-CSP-12	6.7	PLA	PVA
	19.1	PALF-CSP-19	13.1	PLA	PVA

a Note: CSP: composite sandwich panels, PALF: pineapple leaf fiber, PVA: polyvinyl acetate, and PLA: polylactic acid.

**Figure 1:**
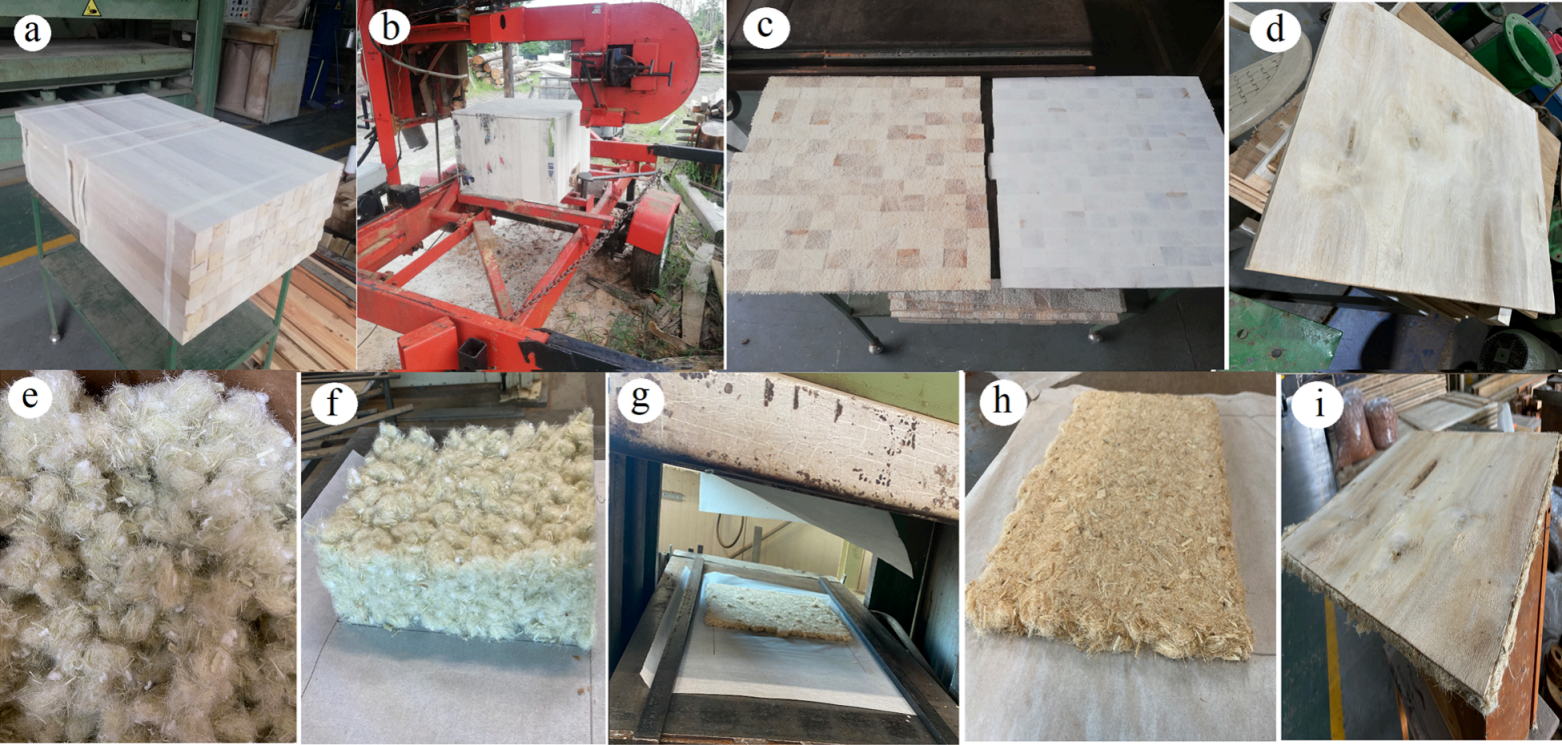
CSP with balsawood (a–d) and PALF (e–i): block fabricated of 80 cm × 80 cm × 100 cm (a), core balsawood sandwich extracted with bandsaw (b), core balsawood without sand and sanded (c), CSP-balsawood (d), PALF cut to 2 cm in length (e), foams of PALF before press (f), foam of PALF after pressed (g, h), and CSP- PALF panels (i). Copyright 2025.

**Figure 2:**
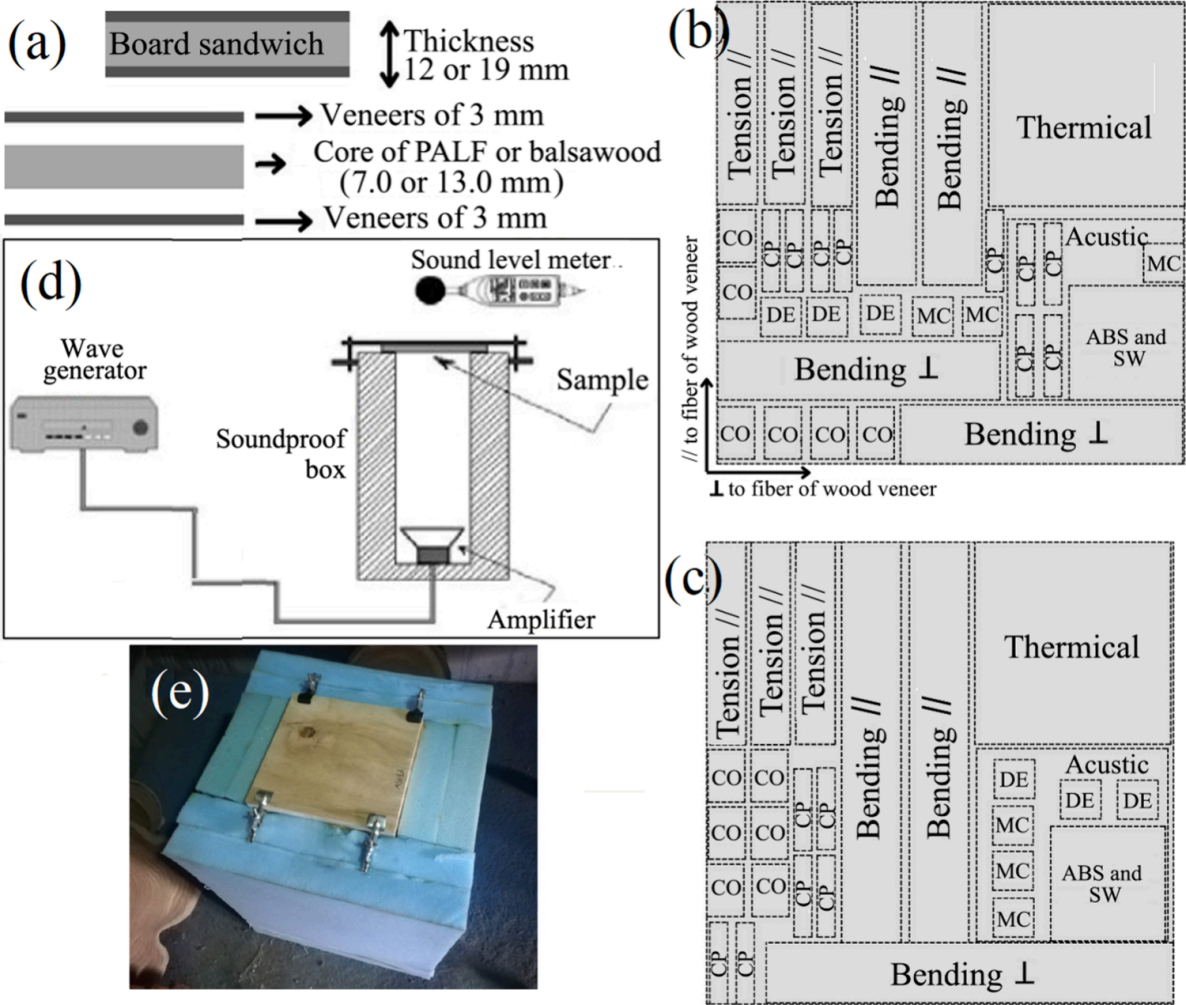
(a) Distribution of *G. arborea* veneers and core of balsawood and PALF in CSP, (b, c) sawn pattern utilized in obtaining of different specimens for properties evaluated, (d) distribution of different components in acoustic test, and (e) soundproof box fabricated for acoustic test. Copyright 2025.

The production process involved an initial stage focusing on creating the core sandwich, followed by a final stage related to CSP production. In the production of the balsawood sandwich core, an end-grain panel was utilized, which is believed to have superior acoustic properties compared to a longitudinal panel.^13^ Each sample of dried balsawood, with a moisture content of 12%, was sectioned to dimensions of 5 cm × 5 cm × 100 cm (width × thickness × length) and glued together with PVA to conform the panel applied on one face of the board with a glue spread rate of 220 g m^–2^ using a glue roller. Seventeen of these individual wood samples were bonded together with PVA glue to form a wood layer measuring 80 cm in width and 5 cm in thickness, which was then smoothed using wide belt sanders (model Sandya1, SCM, Italy). Subsequently, 17 wood layers were pressed to reach a block (Figure 1a) size of 80 cm × 80 cm × 100 cm (length × width × thickness) with a specific pressure of 30 Pa at room temperature, approx 22 °C. From this block, 10 sandwich boards, each measuring 6.3 cm, and another 10 boards, each measuring 13.1 cm in the transverse direction, were extracted (Figure 1b). The core balsawood sandwich was then sanded using an SCM Sandya 1 wood caliper until the desired thicknesses were achieved (Figure 1c).

In the case of PALF, the fibers were extracted from the leaf, dried, and cut to a length of around 2 cm (Figure 1e), ensuring that they were well-separated to prevent any entanglement. Using a blender, 8% PLA was incorporated by weight relative to the total fiber weight. Overall, 10 core sandwiches measuring 60 cm × 60 cm were produced, with five cores having a thickness of 6.3 cm and the other five at 13.1 cm (Figure 1i). The target density for these core sandwiches was set at 100 kg m^–3^ after press (Figure 1g,h). The foams (Figure 1f) underwent a pressing process at 30 Pa and 175 °C for 10 min and were then placed in a climate-controlled chamber at 22 °C and 66% relative humidity for 24 h. This step was crucial for balancing the moisture content and completing the PLA curing process. Finally, it was verified that all core boards of balsawood and PALF did not present any voids with the objective of preventing structural failures before the mechanical test.

The subsequent phase of the process involved creating the CSP, which had *Gmelina arborea* veneers affixed to each side of a core sandwich made from either balsawood or PALF (Figure 1d,j). For each veneer, 127 g of PVA adhesive was used, with the veneer weighing 353 g m^–2^. The balsawood core sandwiches were pressed under a specific pressure of 60 Pa at a temperature of 90 °C for roughly 40 min. In contrast, panels with a PALF core were subjected to a specific pressure of 30 Pa at the same temperature of 90 °C for about 40 min. To prevent applying too much pressure on the panels, stops were created to achieve the desired panel thickness. Afterward, all panels were stored for a week at a temperature of 22 °C and a relative humidity of 66%, achieving an equilibrium moisture content of 12%.

### Sampling Methods

Five CSP units were produced and cut following the pattern illustrated in Figure 1f,g, with the aim of extracting all of the necessary test specimens. Density (DE), water absorption (WA), moisture content (MC), and thickness swelling (WT) for assessing physical properties and parallel compression (CP), parallel tension (TP), flexure in the parallel direction (FP), flexure in the perpendicular direction (FP), and shear (SH) for mechanical properties. Tests for thermal conductivity and acoustic insulation were also conducted. For each variable, 10 specimens were extracted according to the pattern shown in Figure 1f,g. For compression and shear tests, three and four individual specimens, respectively, were glued with PVA to achieve the minimum 4–5 cm required thickness.

### Composites Sandwich Panel Evaluation

#### Physical and Mechanical Properties

The physical properties of CSP were tested according to the following American Society for Testing and Materials (ASTM) standards D4442–20 ^34^ for MC, ASTM D2395–17 ^35^ for DE, and ASTM D1037–12 ^36^ for WA and WT. Mechanical properties were determined following ASTM D1037–12 ^36^ with a Tinius Olsen model H10KT (PA, California, USA) universal testing machine.

#### Acoustic Insulation Test

For conducting this CSP test, sound attenuation parameters were assessed at various sound power levels utilizing a wave generator, an amplifier, and a sound level meter according to the methodology proposed by González et al.,^37^ which they adjusted the distance of the panel from the amplifier proposed by Parbrook et al.^38^ To achieve this, a soundproof box with one open side was constructed with the amplifier connected to a wave generator positioned outside at a minimum distance of 30 cm from the bottom. Additionally, the sound level meter was placed outside the box at 30 cm from the specimen board (Figure 1h). The soundproof box was fabricated using 12 mm oriented strand board (OSB) panels and lined with 10 cm thick polyurethane foam (Figure 1i). To ensure optimal insulation, the sample was mounted perpendicular to the frame and securely supported. Extech 407780 sound level meter from Nashua, New Hampshire, USA, was used.

Five repetitions were conducted for each CSP type. The sound level meter assessed the decibel levels from 30 cm from the panel across six wave frequencies of 250, 500, 1000, 2000, 4000, and 8000 Hz. Furthermore, the decibel levels were recorded without panel samples, referred to from now on as control values. A commercial acoustic foam made from plastic fibers and polymers originating from Costa Rica was also tested to provide a comparative analysis, from now on referred to as commercial products. The sound level meter measured the noise in decibels (dB), after which the sound transmission loss (STL), sound transmission coefficient (STC), and sound absorption coefficient (SAC) were calculated for each frequency and subsequently averaged for the CSP.

Sound transmission loss (STL) is determined by the difference in sound intensity in decibels (dB) without any insulating material or control compared to the intensity in dB measured when using the specimens, as indicated by eq 1. A higher STL value signifies greater sound reduction due to the board. Meanwhile, the sound transmission class (STC) is calculated as the average of the STL values for each sample or material, as shown in eq 2. The noise reduction coefficient (NRC) is the average of the sound absorption coefficient (SAC) values across all frequencies for a given sample or material. The SAC is derived using eq 3, which compares the values obtained from STL to the values from the noise power spectrum (NPS) when no insulator is present. In such cases, the expected SAC value is 1, as there is no barrier to impede sound reduction.

$$\mathrm{STL}=I_{\mathrm{control}}-I_{\mathrm{panels}\;\mathrm{sandwich}\;\mathrm{type}}$$ 1

$$\mathrm{STC}=\frac{\sum_{1}^{N}\mathrm{STL}}{N}$$ 2

$$\mathrm{SAC}=\mathrm{NRCideal}\times \frac{\mathrm{STL}}{\mathrm{NPS}}$$ 3

where STL is the sound transmission loss in dB, STC is the sound transmission loss in dB, SAC is the sound absorption coefficient in dB, NPS is the frequency of control in dB, and NRCideal is the ideal sound reduction coefficient, which in this case represents a value of 1.

#### Thermal Conductivity

The thermal conductivity of 25 × 25 cm samples was measured using an HFM 446 M, Lambda Eco-Line heat flow meter from the NETZSCH Group in Selb, Germany, at temperatures of 10, 20, and 30 °C. This thermal conductivity evaluation adhered to the UNE-EN 12667 standard.^39^ The testing setup involved a single-sample device in which one sample was substituted with a combination of an insulation piece and a protective plate. Each board required approximately 3 h for testing, with a heating rate of 1 °K min^–1^. Thermal analysis encompasses a set of analytical techniques that provide insights into materials by observing changes in structure and properties due to temperature fluctuations^27,29^ and was carried out using the Origin­(Pro) software Version 2022.^40^

### Data Analysis

Data analysis involved general statistics, such as the average and the coefficient of variation. The normality of the data and any outliers were identified. If such values were detected, then they were removed and replaced with the average of the measurements. Subsequently, an analysis of variance (ANOVA) was conducted to identify significant differences in the properties evaluated across the various sandwich composite board treatments. Additionally, a Tukey test with a *P*-value of less than 0.01 was implemented to confirm significant differences between the means of each board thickness. The statistical analysis was carried out using the InfoStat software 2014 from the National University of Córdoba in Córdoba, Argentina, which presents the advantage of being a free software used in Latin America.^41^

## Results

### Physical and Mechanical Properties

The physical characteristics of CSP are outlined in Table 2. CSP constructed with a PALF core tended to be about 2 mm thinner than those made with a balsawood core. The moisture content was consistent across all thicknesses and aligned with the target moisture content under stabilization conditions, which is 12%.

**Table 2:** Physical Properties for CSP with Balsawood or PALF as the Core^a^

	type of composite sandwich panels			
physical properties	PALF-CSP-12	PALF-CSP-19	Balsa-CSP-12	Balsa-CSP-19
thickness (mm)	10.5 (0.83)^D^	16.5 (1.01)^B^	12.5 (0.47)^C^	18.6 (0.68)^A^
moisture content (%)	12.3 (0.31)^A^	11.3 (0.50)^B^	12.7 (0.53)^A^	12.9 (0.13)^A^
water absorption (%)	103.6 (11.72)^A^	127.7 (13.87)^A^	60.0 (11.33)^B^	69.00(10.38)^B^
swelling in thickness (%)	8.52 (2.49)^A^	3.40 (0.52)^B^	1.53 (0.48)^B^	0.92 (0.29)^B^

a Values in parentheses mean standard deviation. Different letters next to standard deviation represent statistical difference at 99% significance by the Tukey test for the same properties in different CSP.

The density of CSP varied based on the type of core and the thickness of each material, as shown in Table 2. The PALF-CSP-12 composite exhibited the highest statistical density value at 303 kg m^–3^, whereas PALF-CSP-19 and Balsa-CSP-19 had the lowest densities, ranging between 210 and 222 kg m^–3^. Balsa-CSP-19 had intermediate density values of 266 kg m^–3^. A significant observation is the density difference: the gap between Balsa-CSP-12 and Balsa-CSP-19 is smaller than that between PALF-CSP-12 and PALF-CSP-19. Moreover, cores made with PALF showed a density between 100 and 110 kg m^–3^, while those made with balsawood ranged from 137 to 141 kg m^–3^. No statistical difference was noted between the two thicknesses for the same material, as illustrated in Figure 3.

**Figure 3:**
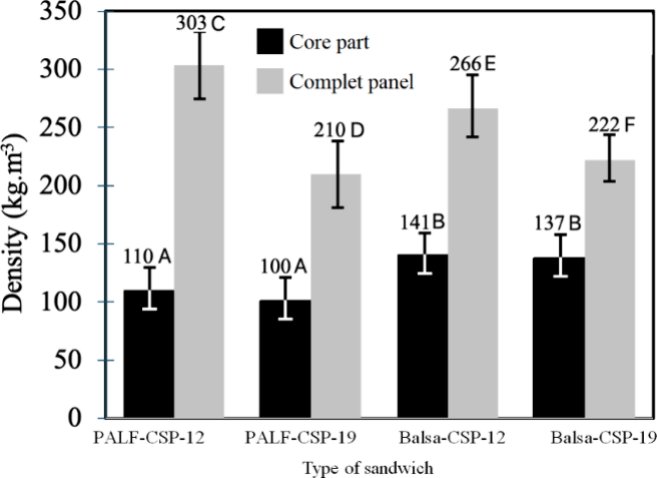
Density of the core sandwich and CSP of balsawood and PALF with acoustic and thermal insulation properties. Note 1: The bars mean standard deviation. Note 2: Letters A and B next to bars for density in core part represent statistical difference at 99% significance by Tukey test in different CSP and C, D, E and F next to bars for density for complete panel represent statistical difference at 99% significance by Tukey test in different CSP.

In relation to WA, it was found that sandwich composites fabricated with PALF presented WA values higher than 100% compared with CSP-balsawood, and no statistical difference was observed between the two different thicknesses. The composites fabricated with balsawood core presented WA values between 60 and 69% with no difference between the two thicknesses (Table 2), but statistically lower than CSP produced with PALF as core.

SW thickness was higher in CSP-PALF than in CSP-balsawood. It was observed that there was a statistical difference between the thicknesses of 12 and 19 mm of the sandwich produced with PALF as core, but any statistical differences were in the two thicknesses of CSP produced with balsawood (Table 2). In general, it was reported that there was no relationship between water absorption and swelling in thickness (Figure 4). However, there was a slight relation if it is considered the same material and same thickness (Figure 4).

**Figure 4:**
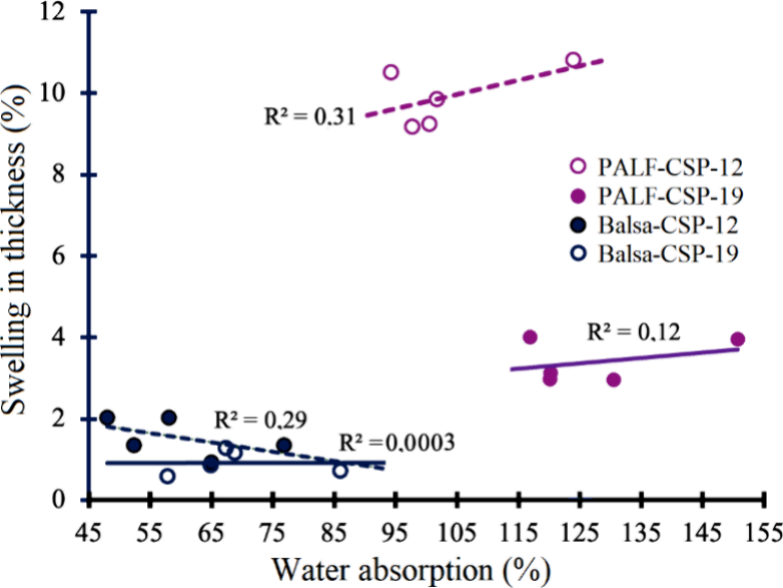
Determination coefficient of water absorption and thickness swelling.

### Mechanical Properties

The findings related to the mechanical properties are detailed in Table 3. The static bending tests conducted in the longitudinal or parallel direction revealed that the MOR and MOE values of the CSP-balsawood exceeded those of the CSP-PALF for both thicknesses. When examining the MOR in perpendicular bending, it was observed that for both core types, the 19 mm-thick samples exhibited lower values compared to the 12 mm samples. Conversely, in parallel bending, CSP-PALF showed that lower thicknesses corresponded to higher values, a pattern not observed in CSP-balsawood (Table 3).

**Table 3:** Mechanical Properties for Different CSP of Balsawood and PALF^a^

		type of composite sandwich panels			
physical properties		PALF-CSP-12	PALF-CSP-19	Balsa-CSP-12	Balsa-CSP-19
perpendicular bending (MPa)	MOR	5.12 (1.10)^A^	2.16 (0.58)^B^	30.55 (3.75)^C^	19.04 (4.13)^D^
	MOE	25.3 (8.37)^A^	9.2 (1.79)^B^	527.0 (35.74)^C^	451.9 (36.01)^D^
parallel bending (MPa)	MOR	0.79 (0.22)^A^	0.48 (0.10)^B^	1.23 (0.20)^C^	1.20 (0.40)^C^
	MOE	5.59 (1.89)^C^	3.63 (1.64)^D^	8.38 (2.77) ^B^	10.75 (2.13)^A^
maximum stress in parallel compression (MPa)		5.97 (1.59)^A^	2.79 (1.36)^B^	9.36 (2.21)^C^	6.06 (1.11)^A^
maximum stress in parallel tension (MPa)		16.50 (4.16)^A^	8.85 (3.16)^B^	16.08 (2.52)^A^	9.30 (2.60)^B^
maximum stress in shear (MPa)		0.007 (0.03)^A^	0.007 (0.03)^A^	0.401 (0.12)^B^	0.425 (0.13)^B^

a Values in parentheses mean standard deviation. Different letters next to standard deviation represent statistical difference at 99% significance by the Tukey test for the same properties in different CSP.

In terms of MOE during perpendicular bending, the 12 mm panels demonstrated higher MOE values than the 19 mm CSP for both PALF and balsawood. When evaluating MOE in parallel bending, although statistical differences were noted among all variables, the trends differed based on the core type. For CSP-PALF, reduced thicknesses resulted in higher values, whereas for CSP-balsawood, the trend was the opposite (Table 3).

In parallel compression and parallel tension tests, the reported values were higher in the CSP-balsawood as core than in those manufactured with PALF for the same thickness. Meanwhile, for the same type of sandwich core, the 12 mm CSP presented a greater maximum stress than that presented in the 19 mm CSP (Table 3).

The evaluation of the parallel tension test reported that, comparing the same thickness, the CSP-PALF as core values were larger than those produced with balsawood. Regarding shear resistance, the sandwiches produced with balsawood presented higher values than those produced with PALF for both thicknesses. If the same type of core is considered, then there were no statistical differences between the two different thicknesses.

#### Acoustic Insulation Test

Figure 5a illustrates that with the exception of the 19 mm balsawood CSP, all samples show a reduction in captured noise between 250 and 500 Hz. Nonetheless, each type of panel begins to enhance noise capture from 500 to 2000 Hz, followed by a decline until it reaches 8000 Hz. A key point to consider is that for the four types of CSP manufactured, the noise capture values are significantly lower than those of the control and the commercial product used for comparison. Regarding the two material types, PALF-CSP-12 exhibits higher solid capture values than PALF-CSP-19. This variance is not observed when comparing panels with balsa as the core material (Figure 5a). When assessing sound transmission and the SAC coefficient, it is noted that the commercial product shows the lowest values, whereas the CSP-balsawood displays higher values than the CSP-PALF (Figure 5b,c).

**Figure 5:**
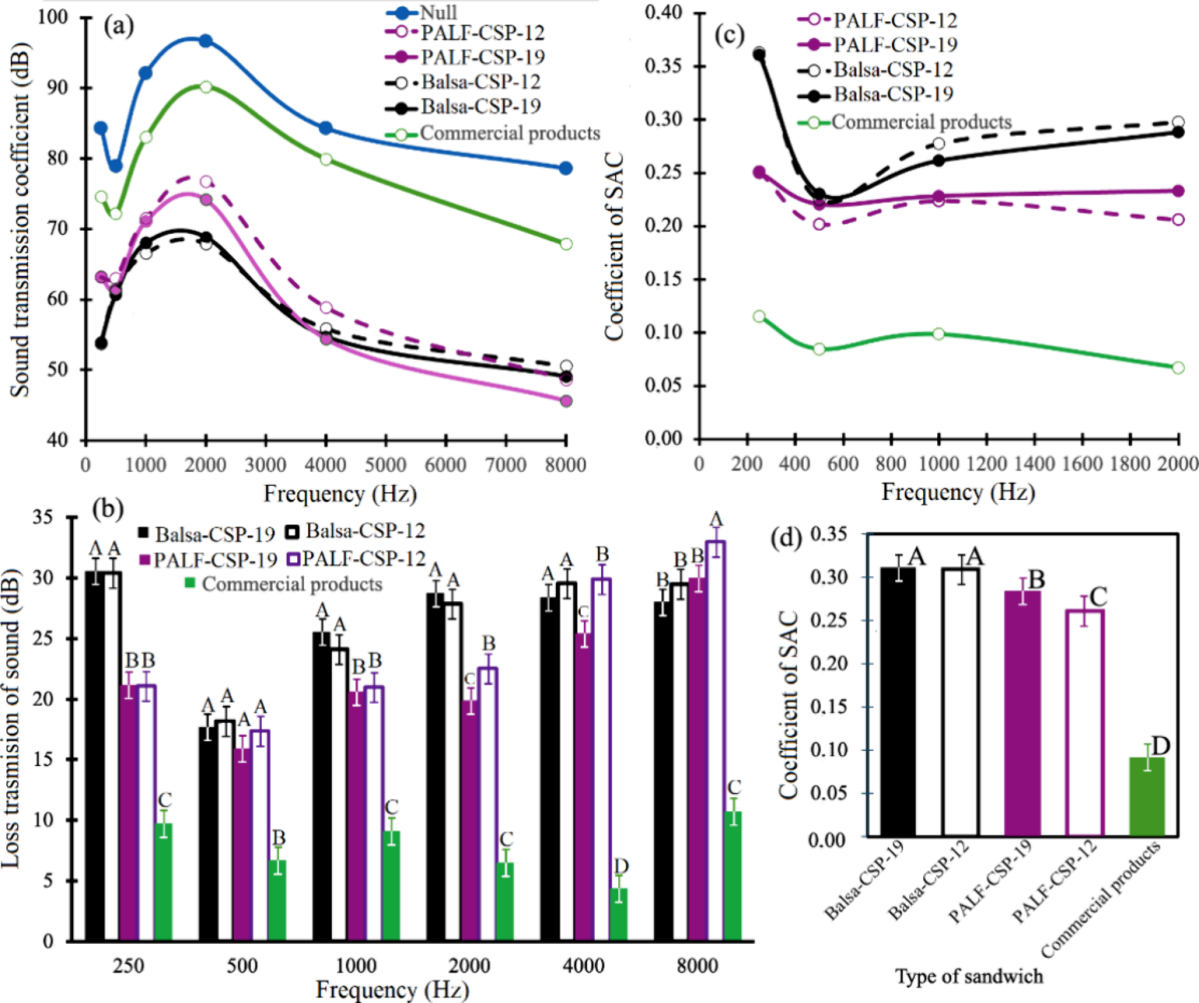
(a) Average noise capture values, (b) sound absorption coefficients (SAC), (c) transmission of sound, and (d) sound reduction coefficients obtained for different CSP of balsawood and PALF with acoustic and thermal insulation properties. Note 1: The bars mean standard deviation in panels (b) and (d). Note 2: Different letters next to bars represent statistical difference at 99% significance by the Tukey test for the same frequency in different CSP in panel (b) and different letters next to bars represent statistical difference at 99% significance by the Tukey test for different CSP.

During the same test, all boards produced demonstrate a similar trend up to a frequency of 500 Hz. However, as the frequency increases to 2000 Hz, the boards made with balsa, for both thicknesses, show higher values. In contrast, for PALF CSP, no statistical difference is noted between the two thicknesses (Figure 5b,c). Overall, the average SAC values across all frequencies reveal that the CSP made with balsawood boasts the highest SAC coefficient. Among the PALF CSP, PALF-CSP-19 has a statistically higher SAC coefficient than PALF-CSP-12 (Figure 5d). It is important to emphasize that both PALF and balsawood CSP have higher SAC coefficient values.

### Thermal Properties

CSP with a balsawood core showed greater thermal conductivity compared to that of CSP with a PALF core throughout the temperature range examined (Figure 6a). When examining different thicknesses, it was found that PALF-CSP-19 exhibited slightly higher thermal conductivity than PALF-CSP-12. Conversely, CSP made with balsawood displayed the opposite results; Balsa-CSP-12 had higher thermal conductivity values than Balsa-CSP-19, with differences more pronounced than those seen in PALF composites (Figure 6a). As anticipated, all cases demonstrated an increase in thermal conductivity as the temperature increased, with a similar gradient (Figure 6a).

**Figure 6:**
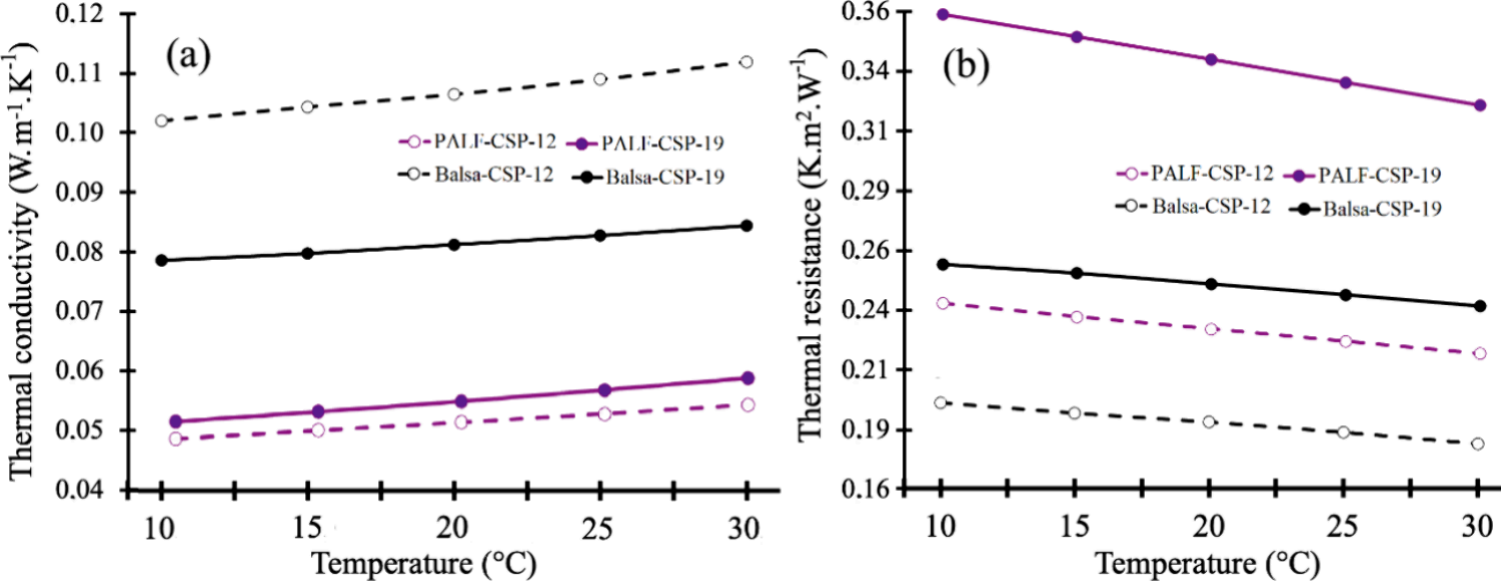
Thermal conductivity (a) and thermal resistance (b) in different temperatures for different CSP values of balsawood and PALF.

In relation to thermal resistance, it was observed that CSP-PALF thermal resistance was higher than CSP-balsawood in the same thickness. In addition, 19 mm CSP in two types of cores (PALF and balsawood) presented higher thermal resistance values than those observed in 12 mm CSP. In addition, the thermal resistance decreased with the increase in temperature in all the variables (Figure 5b). The weather condition is 25 °C in many tropical areas, and then the results of thermal conductivity and thermal resistance are presented at 25 °C (Table 4). Another important observation was that panel density affected thermal properties was related to density. For example, CSP fabricated with PALF in a thickness of 10 mm (with the highest density) presented lower thermal conductivity values than 19 mm (with the lowest density). However, for CSP fabricated with balsawood, the highest thermal conductivity was observed in CSP with the highest density, contrary to results for CSP fabricated with PALF (Table 4).

**Table 4:** Thermal Conductivity and Thermal Resistance at 25 °C of Temperature for Different CSP of Balsawood and PALF

type of board sandwich	thickness (mm)	density (kg m^–3^)	thermal conductivity (W m^–1^ K^–1^)	thermal resistance (K m^–2^ W^–1^)
PALF-CSP-12	10	334.02	0.05406	0.22193
PALF-CSP-19	17	249.65	0.05754	0.33021
Balsa-CSP-12	12	293.19	0.10907	0.18336
Balsa-CSP-19	19	189.21	0.08285	0.24240

### SEM Observations in Glue Line

The SEM observation of the core of CSP showed that bundles of PALF in the core were glued between them by PLA adhesive, and it was observed that some fiber of PLA was not melted (Figure 7a), probably because this part of the panels did not reach the appropriate temperature. Meanwhile, the SEM observations of the glue line between *G. arborea* wood and the core of PALF presented a thickness between 160 and 180 μm, and the depth of the adhesive was limited (Figure 7b). On the other hand, the glue line formed by veneers of *G. arborea* and core of balsawood showed that thickness varied from 160 to 190 μm in two different side of panel: (i) cross section of veneers and longitudinal section of balsawood (Figure 7c) and (ii) cross section of veneers and longitudinal section of balsawood (Figure 7d).

**Figure 7:**
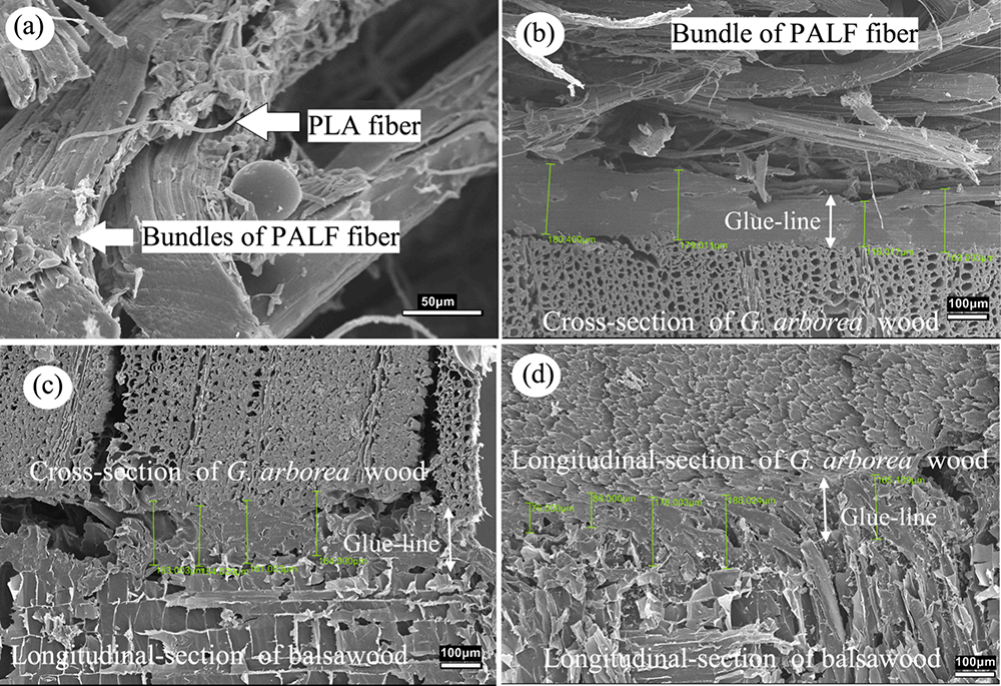
Scanning electron microscopy (SEM) photograph of CSP fabricated with PALF (a, b) and balsawood (c, d). Bundles of PALF glued with PLA (a), glue line formed by PALF and the veneer of *G. arborea* wood in the cross section (b), glue line formed by the veneer of *G. arborea* wood in the cross section and balsawood in the longitudinal section (c) and glue line formed by the veneer of *G. arborea* wood in the longitudinal section and balsawood in the longitudinal section (b). Copyright 2025.

## Discussion

### Physical and Mechanical Properties

Sound and temperature insulating panels are available in a broad range of densities.^25^ However, to qualify as insulators, panels must have a low density, usually less than 200 kg m^–3^.^42^ They must adhere to suitable thermal and acoustic conductivity standards, which means the insulation materials should use porous fibers, as this enhances their insulating capabilities for both heat and sound.^43^

Consequently, although CSP produced with balsa and PALF cores and melina wood veneers have overall product densities exceeding 200 kg m^–3^ (Figure 3), the cores themselves maintain a relatively low density, under 150 kg m^–3^, thus fulfilling the insulating criteria by exhibiting an appropriate density. The rise in CSP density results from the wood veneer utilized, which possesses a density ranging from 400 to 450 kg m^–3^, leading to an increase in the CSP’s total density.^30^ An additional key point is that the cores crafted with these two materials exhibit minimal density variation (Figure 3), offering the benefit that the potential final products will display consistent properties. Therefore, balsawood and PALF cores with consistent density will not largely impact the CSP structure’s performance, unlike when there is a significant density variation within the core.^13^

While CSP exhibit a generally high density, wood-based insulation panels with densities similar to those in this study have been documented. For instance, Gößwald et al.^44^ identified densities ranging from 160 to 300 kg m^–3^ in insulation boards produced with spruce bark fibers. However, when CSP is produced using alternative materials like oil plant trunk, densities can exceed 600 kg m^–3^, still demonstrating excellent insulation properties.^45^ Focusing on the core alone, the density of panels made with balsawood and PALF aligns with many low-density CSP composed of foams or lignocellulosic wools.^27,29^ These materials are known for their porous nature and lumens, providing suitable thermal and acoustic conductivity.^43^

When each core type is examined in comparison to other research, lower density outcomes are noted. For example, Zhang et al.^46^ reported a CSP with a steel sheet and balsawood core density of 160 kg m^–3^. Similarly, Osei-Antwi et al.^47^ found a considerable range in density for end-grain balsa wood sandwiches, from 180 to 252 kg m^–3^, which matches the density range identified in this study. Lastly, Zuccarello et al.^48^ created CSP with a core density of 150 kg m^–3^ using balsa and various materials for the core faces, exhibiting values comparable to the balsawood core density in the current study.

In the study conducted by Puttra and colleagues in 2018, the density of CSP made with PALF was found to range from 50 to 150 kg m^–3^. Ali and his team^49^ reported densities between 122 and 161 kg m^–3^, while Thilagavathi et al.^50^ noted that PALF composites had core densities ranging from 194 to 200 kg m^–3^. However, when poly­(ethylene terephthalate) (PET) is incorporated, the density rises to 240 kg m^–3^. The density values for the PALF sandwich core examined in the current study align closely with those reported in these earlier studies.

Examining the influence of thickness on density, it was observed that the thinnest boards exhibited the highest density values for identical materials (Figure 3). This phenomenon occurs because the density is affected by the thickness of the *Gmelina arborea* veneer and its contribution to the board’s overall thickness. Melina wood has a density near 400 kg m^–3^,^30^ while the cores made of PALF and balsawood are under 200 kg m^–3^ (Figure 3). Therefore, in the thinner CSP, the wood veneer constitutes a larger portion of the thickness compared to CSP that are 19 mm or thicker. This results in an increased density for the 12 mm CSP due to the greater proportion represented by the wood veneer.

The water absorption rates were notably high across all types of CSP, with CSP-balsawood showing absorption levels over 60% and CSP-PALF exceeding 100% (Table 2). These findings are consistent with the absorption values observed in other natural fibers used for thermal insulation CSP or wood-based construction materials.^51^ Natural fibers inherently tend to absorb humidity and water, which can promote conditions favorable for fungal growth that may lead to core degradation.^52^ In this particular study, it was noted that CSP made with PALF exhibited a significant amount of swelling compared with those using balsawood as a core, highlighting a disadvantage of PALF in comparison to balsawood.

The variation in WA values between the two materials arises from their distinct structures, with flow characteristics influenced by factors like molecular structure, polarity, crystallinity, and the hardeners used in forming composites.^52^ Balsa, despite being porous,^13,53^ allows water to pass through at a slower rate compared to the PALF core, which is even more porous and thus exhibits greater hygroscopicity than balsawood. Nevertheless, the pronounced hygroscopic nature of both balsawood and PALF can be mitigated through various treatments designed to reduce water absorption, thereby lowering TS.^7^ Achieving a sufficient level of water repellency is crucial to ensure that panels effectively resist moisture and retain their insulation capabilities. Therefore, it is essential to minimize water absorption and maintain strong water repellency to prevent moisture from compromising the insulation, which could lead to decreased thermal efficiency, mold development, or structural deterioration.^54^

The resistance values of mechanical properties, such as bending, compression, tension, and shear, have a positive correlation with the density of the materials.^55^ The density of a material is closely linked to the effectiveness of its mechanical properties.^55^ Nonetheless, in the context of CSP, mechanical resistance is influenced not only by density but also by additional factors, highlighting the distribution and characteristics of the materials.^56^ The CSP mechanical property values usually reflect this pattern. For instance, CSP made with balsawood in both 12 mm and 19 mm thicknesses exhibited higher values in bending, compression, and shear, despite having lower densities compared to those produced with PALF (Figure 3). During the tension test, it was noted that resistance behavior does not correlate with thickness. Both balsawood and PALF cores of the same thickness reached maximum stress values without any statistical difference. (Table 3). The findings indicate that the resistance of the CSP is influenced by the type of core used during the production of the CSP. Specifically, the PALF-CSP has a lower density compared to CSP that uses balsawood as a core (Figure 3). Consequently, it exhibits reduced resistance in bending, compression, and shear evaluations. This difference between the two different cores is due to the two materials presenting different anatomical and chemical structures, crystallinity, and the hardeners used in forming composites.^52^ Then, balsawood is a 3D hierarchically porous cellular structure more strongly linked than the link presented in the PALF core. So, the densely packed closed fibers in balsawood provide mechanical support for enhanced strength.^57^

The PALF core is composed of a cluster of fibers embedded in foam and combined with synthetic adhesives or bioplastics like PLA, as noted in this study.^58^ According to Kotteesvaran et al.^59^ one drawback of natural fibers is their weak surface bonding with the composite matrix, which generally diminishes their physical resistance and stability. The presence of waxes in the fiber cell walls and their hydrophilic nature led to poor adhesion. This structure includes several spaces aimed at lowering the foam’s density, which in turn limits the resistance of the foams^56^ and subsequently affects the composite materials, as demonstrated in bending, compression, and shear evaluations (Table 3).

Balsawood exhibits a hierarchical composition, characterized by both tubular and fibrous structures. It is composed of a series of natural polymers that endow it with exceptional mechanical strength,^60,61^ often surpassing the resistance and adhesion properties of foams produced with PALF. However, during tension force, the influence of the core is minimal compared to factors such as thickness and the proportion it constitutes of the overall thickness. The panels were fabricated with 3 mm veneer sheets on each side, contributing to their enhanced resistance in tension tests. This is largely due to wood’s high tensile strength in the longitudinal direction, and this configuration should not reveal the true impact of the tensile resistance of the PALF or balsawood core. This suggests that melina wood significantly enhances the CSP, highlighting that PALF or balsawood cores have limited tensile resistance. This underscores the significance of employing panels made of diverse materials, particularly on surfaces that augment certain properties of the boards and mitigate fatigue in sandwich panels.

Typically, it has been found that sandwich panels with either a PALF core or a balsawood core exhibit higher densities in the 12 mm CSP compared to those in the 19 mm CSP (Figure 3). Generally, aside from the maximum shear stress, CSP made with balsawood demonstrates superior mechanical properties (Table 3). This offers a benefit in applications where mechanical characteristics are crucial alongside acoustic and thermal properties.

### Acoustic Assessment

The various parameters assessed in the acoustic properties, such as captured noise, sound transmission loss, and SAC coefficients, indicated that CSP utilizing balsawood as the core generally exhibited superior insulating capabilities. Specifically, they demonstrated low noise capture (Figure 5a), a high SAC coefficient across different frequency levels (Figure 5b), and elevated values of sound transmission loss (Figure 5c) along with a high SAC coefficient (Figure 5d). Notably, both CSP-PALF and CSP-balsawood showed better insulation properties when compared with a synthetic commercial insulation product available in Costa Rica (Figure 5). These CSP excelled in sound absorption due to the multiscale architecture of the fibers on the panel surfaces and the fibrous or balsawood microstructure of the core, thereby offering enhanced performance over other types of synthetic alternatives.^46^ This indicates that CSP made from PALF or balsawood are not only more environmentally friendly but also more efficient than synthetic materials.

The second point to emphasize is that the optimal performance of the manufactured panels starts at 2000 Hz, because at lower frequencies, noise is absorbed and the sound absorption coefficient (SAC) reaches its peak (Figure 5a,b). To grasp this phenomenon, it is essential to comprehend the sound absorption mechanism in composite sandwich panels (CSP) with natural material cores.^46,62^ Initially, the sound energy that hits the panel is partially reflected by the veneer on one side, specifically the *Gmelina arborea* veneer in this instance. The remaining sound energy is transmitted into the interior of the panel in two parts: one part is vertically reflected due to viscous resistance, thermal exchange, and the damping effects of fibers or microfibrils and multilayer cell walls. The other part of the sound energy penetrates further into the core, where a portion is again reflected by the interface between the veneer and the core. The remaining energy is absorbed mainly by the balsawood or PALF core, primarily through viscous resistance, thermal exchange, and the damping effects of fibers or microfibrils and multilayer cell walls, where significant dissipation occurs. The sound energy that remains continues to the other side of the panel, where it is once more reflected by the core-veneer interface and the veneer itself.

When sound energy is lost, various physical phenomena come into play, especially in porous regions (areas with pores and empty spaces) and in the cell wall. Sound waves cause air molecules to vibrate periodically, and the friction between these air molecules and the cell wall generates frictional heat. Additionally, the air within the cell undergoes compression and expansion, leading to a deformation of the cell wall and consequently producing thermal energy.^63^ At low frequencies, such as the case at 2000 Hz, thermal exchange primarily governs energy dissipation. This results in reduced noise capture and consequently low values of SAC. Conversely, at high frequencies, the viscous resistance of the cell wall and the resonance absorption mechanism lead to air molecules vibrating, with the friction between air molecules and the cell wall producing heat.^46,62^

The acoustic characteristics measured for CSP made with PALF and balsawood (Figure 5) were consistent with findings from other research involving different fibers. For instance, Zhang et al.^46^ created three varieties of sandwich structure composites using diverse fibers (including flax-fiber-reinforced composite, flax-fiber-reinforced composite, and flax-fiber-reinforced composite) along with balsawood, reporting sound absorption coefficient (SAC) values ranging from 0.1 to 0.4. This range is similar to what was observed for CSP made from balsawood and PALF (Figure 5d). According to Vladimirova and Gong,^10^ an SAC coefficient above 0.2 is indicative of good insulation properties. The composite sandwich panels made with balsawood or PALF showed coefficient values between 0.25 and 0.3e (Figure 5e), which are notably higher than those reported by Vladimirova and Gong.^10^ Remarkably, these coefficient values also surpass those of commercial insulation panels commonly used in Costa Rica (as shown in Figure 5e), indicating that the CSP constructed with PALF and balsawood demonstrates excellent acoustic performance for panel applications.

### Thermal Assessment

The thermal properties of a material are influenced by several factors, including its density, composition, and distribution.^64^ The thermal conductivity of a material has a direct relation with its density and with the temperature it is exposed to, meaning that higher density and higher temperatures usually lead to higher thermal conductivity.^28,29,65^ Modern construction materials with low thermal conductivity values are typically below 0.1 W m^–1^ K^–1^, and those with values under 0.07 W m^–1^ K^–1^ are classified as thermal insulators.^25^ A material can be deemed a thermal insulator if its thermal conductivity coefficient is less than 0.060 W m^–1^ K^–1^, demonstrating the effective thermal insulation provided by the manufactured pineapple fiber boards.

Balsawood, in particular, is recognized as a solid material with low thermal conductivity, which makes it suitable for providing mechanical support to internal structures.^66^ The thermal conductivity of solid wood is around 0.1 and 0.2 W m^–1^ K^–1^, while the thermal conductivity of a PALF board with a density of 338 kg m^–3^ was 0.057 W m^–1^ K^–1^.^67^ In that sense, Table 4 presents the results for all samples, reporting lower thermal conductivity values for CSP produced with PALF as a core. In the same line, if we compare the density in CSP produced with the same core type, it can be noticed that the higher the core proportion, the lower the density. This value corresponds to 52 and 68% for the thinner (12 mm) and thicker (19 mm) CSP samples, respectively. The porosity of the material is also determinant in the thermal conductivity as more air can retain more heat,^68–70^ highlighting the better insulation properties of the CSP-PALF than CSP-balsa. In the case of CSP-balsa, the higher thermal conductivity and the lower thermal resistance are related to the higher density. However, in the case of CSP-PALF, a higher density is not related to a higher thermal conductivity, but with lower thermal resistance (Figure 6). According to Asdrubali et al.,^71^ low values of thermal conductivity less than 0.1 W m^–1^ K^–1^ are considered thermal insulators. Then, CSP fabricated with PALF and balsawood in two different thicknesses were good thermal insulators because they presented a thermal conductivity less than 0.1 W m^–1^ K^–1^ (Table 4).

While exploring the relationship between acoustic and thermal properties, and other evaluated properties across different thicknesses and materials in the core, it was determined that although varying sample quantities were tested without corresponding samples, a relationship could be established in CSP made with PALF. The PALF-CSP-19 mm panels demonstrated the lowest thermal resistance and SAC coefficient (Table 4), alongside the weakest mechanical properties, except for maximum shear stress. This variation is linked to density; CSP-PALF-19 displayed the lowest density, leading to the expectation that panels of this thickness would exhibit the weakest mechanical properties,^53,55^ lowest thermal resistance, and lowest SAC coefficient, given the association with low density.^46,72^ Nonetheless, the results did not establish a relationship among mechanical, acoustic, and thermal properties in CSP made with balsawood. The Balsa-CSP-12 showed the highest thermal conductivity (Table 4), yet no differences were noted in acoustic properties, while some mechanical properties (bending, compression, and shear stress) were the highest at 12 mm thickness, with the lowest water absorption and swelling values. CSP made with balsawood materials presents a complex hierarchical composition, characterized by tubular and fibrous structures^63^ that lie perpendicularly between the balsawood core and G. arborea veneers. This relationship is influenced by the core’s thickness, the proportion of this thickness in relation to the total thickness, and its density. These results are explained by the focus of the study, which was not on determining the relationship between physical and mechanical properties with thermal or acoustic properties.

The findings reveal that the thermal properties, specifically conductivity, are less than 0.1 W m^–1^ K^–1^ for CSP with balsawood and under 0.060 W m^–1^ K^–1^ for CSP with PALF. Additionally, SAC values exceed 0.2 for both types of CSP, indicating excellent insulation capabilities in terms of both sound and thermal insulation. The panels are characterized by low density, minimal swelling, and commendable mechanical properties, making them suitable for fabrication into panels that can be applied in various systems. These panels can enhance energy efficiency, improve spatial conditions, and reduce energy consumption, thereby aiding in climate change mitigation efforts.

## Conclusions

Using CSP with balsawood or PALF as a core and *Gmelina arborea* as veneers on their sides offers a promising, sustainable alternative for construction. These panels could enhance energy efficiency and sound dissipation, resulting in more comfortable spaces. The CSP were crafted with thicknesses of 12 and 19 mm, employing PVA adhesive for solid wood components and PLA as a natural binder for PALF.

The panels showed density values below 300 kg m^–3^, classifying them as low-density materials. However, they exhibited high water absorption rates, exceeding 100% for PALF and 60% for balsawood. The primary benefit of these panels is their core, made of renewable and low-density materials; in addition, *Gmelina arborea* veneers were added on the sides, providing a significant improvement in the strength. While the cores of PALF or balsawood offer limited resistance, the use of varied materials, especially on the faces, enhances certain properties and improves the durability of the sandwich-type panels.

CSP-balsawood offered superior performance as an acoustic insulator. They showed low noise capture, high SAC coefficient at various frequency levels, and significant sound transmission loss and SAC values (values over 0.2 are considered good insulation properties according to literature reports^73^); then, composite sandwich panels of balsawood or PALF improve the acoustic performance of panels. Panels made with PALF exhibited a slightly lower acoustic performance as acoustic insulators. Nevertheless, both PALF and balsawood cores performed better in acoustic insulation than commercial synthetic insulation products available in Costa Rica. The thermal conductivity at 20 °C of all the CSP presented acceptable values, remaining below 0.09 W m^–1^ K^–1^, with the exception of Balsa-CSP-12 with 0.10907 W m^–1^ K^–1^, qualifying them as effective thermal insulators.

Summarizing, CSP made with balsawood is suitable for applications requiring sound insulation, while those fabricated with PALF are ideal for situations where thermal insulation is a priority.

## Data Availability

Data Availability Statements are available in Repositorio TECdatos at 10.18845/RDA/PZO1RZ.

## Appendix A

### Supplementary material

The online version does not contain Supporting Information.
